# Homodimerisation-independent cleavage of dsRNA by a pestiviral nicking endoribonuclease

**DOI:** 10.1038/s41598-018-26557-4

**Published:** 2018-05-29

**Authors:** Carmela Lussi, Kay-Sara Sauter, Matthias Schweizer

**Affiliations:** 1 Institute of Virology and Immunology, Laenggass-Str. 122, CH-3001 Bern, Switzerland; 2 Department of Infectious Diseases and Pathobiology, Vetsuisse Faculty, University of Bern, Bern, Switzerland; 3 Graduate School for Cellular and Biomedical Sciences, University of Bern, Bern, Switzerland; 4 Present Address: Department of Clinical Research, Faculty of Medicine, University of Bern, CH-3010 Bern, Switzerland

## Abstract

The glycoprotein E^rns^ plays a central role in the biology of the pestivirus bovine viral diarrhea virus (BVDV). This soluble endonuclease mediates the escape from an interferon (IFN) response in the infected fetus, thereby permitting the establishment of persistent infection. Viral single-stranded (ss) and double-stranded (ds) RNA act as potent IFN inducing signals and we previously showed that E^rns^ efficiently cleaves these substrates, thereby inhibiting an IFN response that is crucial for successful fetal infection. Considering that a large variety of RNases and DNases require dimerisation to cleave double-stranded substrates, the activity of E^rns^ against dsRNA was postulated to depend on homodimer formation mediated by disulfide bonds involving residue Cys171. Here, we show that monomeric E^rns^ is equally able to cleave dsRNA and to inhibit dsRNA-induced IFN synthesis as the wild-type form. Furthermore, both forms were able to degrade RNA within a DNA/RNA- as well as within a methylated RNA/RNA-hybrid, with the DNA and the methylated RNA strand being resistant to degradation. These results support our model that E^rns^ acts as ‘nicking endoribonuclease’ degrading ssRNA within double-stranded substrates. This efficiently prevents the activation of IFN and helps to maintain a state of innate immunotolerance in persistently infected animals.

## Introduction

Bovine viral diarrhea virus (BVDV), a pestivirus within the family *Flaviviridae*, occurs throughout the world with far-reaching consequences for animal health and the agricultural economy. Infection of pregnant cows within the first ∼120 days of gestation with a noncytopathic (ncp), but not cytopathic (cp), biotype of BVDV may result in the birth of persistently infected (PI) calves^1–6^. The early time point of fetal infection prior to the development of adaptive immunity and the distinct epitheliochorial placenta of ruminants that is impermeable to antibodies, however, cannot fully account for the successful establishment of persistent infection, because the innate immune defense is operative from the earliest time of fetal development. Therefore, in addition to the establishment of virus-specific B- and T-cell immunotolerance during ontogeny and to the lack of transfer of maternal antibodies to the fetus, the interplay of BVDV with the innate immune response of its host animals might be the most important aspect for the long-term survival of this virus in its host population (for review, see refs^1–6^).

Various pattern recognition receptors (PRR), such as the membrane-bound Toll-like receptors (TLR) or the cytoplasmic RIG-I-like receptors (RLR), activate the synthesis of interferon (IFN) type-I (IFN-α/β) upon detection of viral pathogen-associated molecular patterns (PAMP)^7–9^. IFN is a cytokine involved in the most important innate defense mechanism against virus infection, and most viruses, including the pestiviruses, have developed an enormous variety of strategies to bypass these antiviral effects^5,10–14^. The genome of the enveloped pestiviruses consists of a single-stranded (ss) RNA of positive polarity encoding for a single large polyprotein. Cap-independent translation is started by an internal ribosomal entry site (IRES), and the polyprotein is then further processed by cellular and viral proteases into four structural and at least eight non-structural viral proteins^15–17^. The N-terminal autoprotease N^pro^ and the structural protein E^rns^ are exclusively present in viruses of the pestivirus genus within the flaviviridae family, and both were implicated to be involved in the evasion of the host’s type-I IFN defense^2,17,18^. N^pro^ activates the proteasomal degradation of the interferon regulatory factor (IRF)-3^19–22^, and possibly also IRF-7^23^, thus preventing the transcriptional activation of the IFN genes. In this manner, the non-structural protein N^pro^ is able in infected cells to prevent IFN synthesis that is induced by the activation of a large variety of PRRs, e.g. RLRs or TLRs. By contrast, soluble E^rns^ is taken up by clathrin-mediated endocytosis into endolysosomal compartments^24^, and by virtue of its RNase activity^25^, potently inhibits IFN expression induced exclusively by the addition of extracellular viral single- (ss) or double-stranded (ds) RNA^24,26–28^ even in uninfected cells.

The pestiviral endoribonuclease E^rns^ is, on the one hand, an envelope glycoprotein present in the virus particle, but on the other hand, it is also secreted into the extracellular milieu^29^ due to its unusual type of membrane association by the amphipathic helix in the C-terminal part^30–33^. In addition, E^rns^ in the virus particle and in virus-infected cells was shown to exist as disulfide-linked homodimer involving residue Cys171^34,35^. Recently, it was demonstrated that mutation of Cys171 indeed abrogates homodimerisation of E^rns^ of BVDV without affecting its ability to cleave poly(U) (poly-uridine) as a model substrate for ssRNA, and with only minimal effects on viral replication *in vitro*^36^. Similarly, homodimerisation of E^rns^ of the pestivirus classical swine fever virus (CSFV) was also not required for replication *in vitro*^37^, but a virulent strain of CSFV with homodimerisation-incapable E^rns^ was considerably attenuated *in vivo*^36^.

Under physiological conditions, most mammalian RNases degrade ss- but not dsRNA^38,39^. However, under certain conditions or upon artificial multimerisation, several types of RNases gain dsRNase activity^39,40^. Notably, bovine seminal RNase is a homodimer covalently linked by two disulfide bonds, and is enzymatically active on ss- and dsRNA^41–43^ resembling the activity of pestiviral E^rns^. The dsRNase Dicer, a class 3 RNase III enzyme that is involved in the generation of short double-stranded siRNA molecules, contains two RNase III domains forming an intramolecular dimer to cleave its dsRNA substrate, whereas class 1 enzymes possess only one RNase III domain and, thus, require homodimerisation for dsRNase activity^44^. Accordingly, several type-II restriction endonucleases require homodimerisation to recognize palindromic sites and cleave both strands of DNA^45^. It is, however, still unknown how the pestiviral RNase E^rns^ is able to degrade ss- as well as dsRNA. For this purpose, we analysed E^rns^ mutants that are incapable of homodimerisation for their ability to cleave dsRNA and to inhibit dsRNA-induced IFN synthesis.

## Results

### Extracellular E^rns^ R171 is a monomer

**Figure 1:**
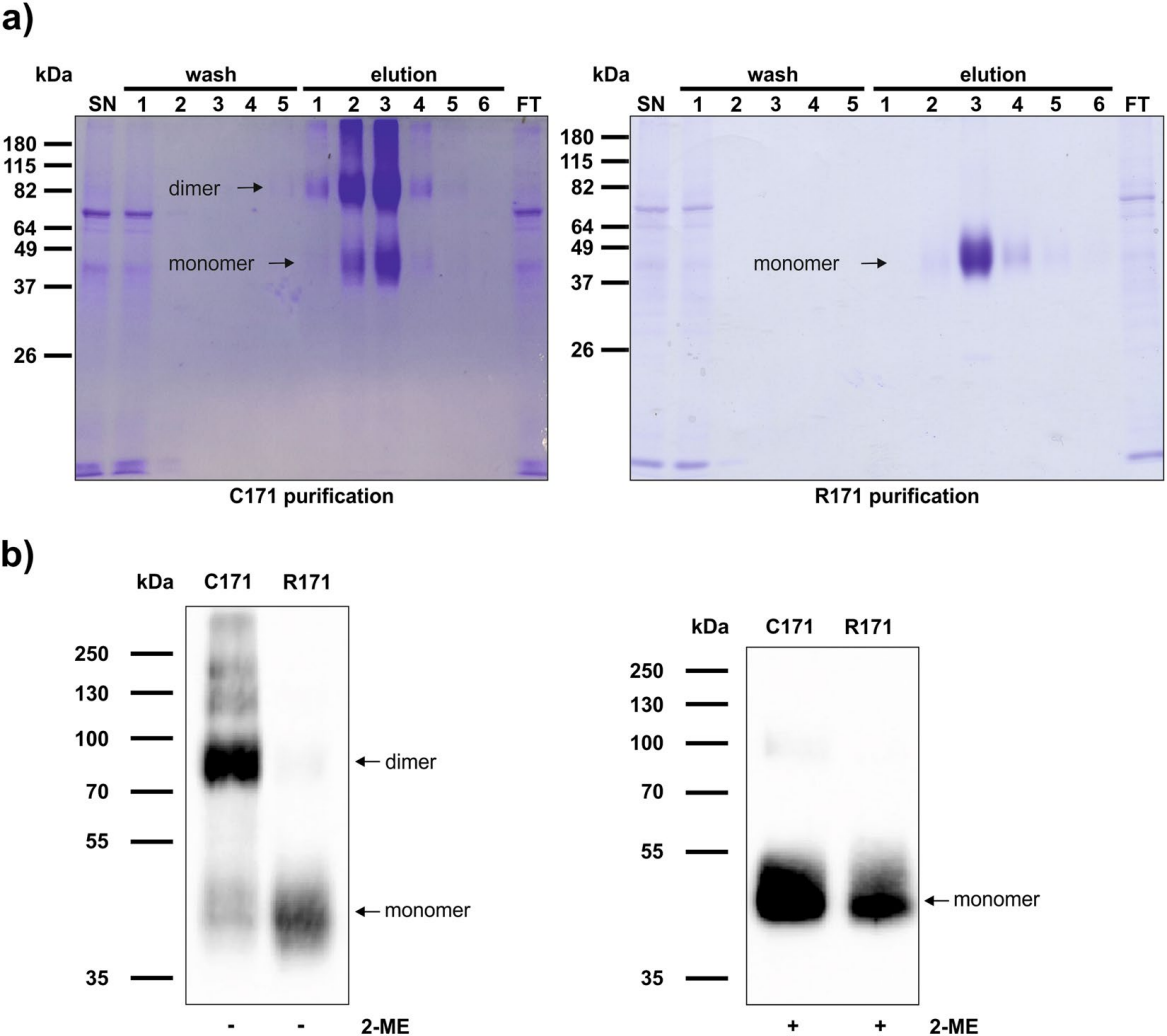
Extracellular E^rns^ R171 is a monomer. (**a**) During the Strep-tag purification process, each sample was collected and equal volumes of the HEK cell supernatant (SN), the five wash steps, the six elution steps as well as the flow through (FT) were separated by SDS-PAGE and stained with Coomassie Brilliant Blue. Purification of the wild-type (C171) and monomeric mutant (R171) are depicted on the left and right panel, respectively. The molecular weights of the proteins are indicated on the left according to the BenchMark™ pre-stained protein ladder. (**b**) Purified, soluble E^rns^ C171 and R171 (2.5 µg each) of the BVDV strain Ncp7 were incubated with or without β-mercaptoethanol (2-ME) as indicated. Samples were analysed by Western blot using an anti-E^rns^ antibody for the non-reducing condition (left panel) or an anti-Strep tag antibody for the reducing condition (right panel). PageRuler Plus pre-stained protein ladder was used for size determination. The signal was detected using a CCD camera with an exposure time of 10 s (left panel) or 5 s (right panel). One representative experiment out of three is shown.

E^rns^ was purified from the supernatant of transiently transfected HEK cells, and its purity was verified by SDS-PAGE and Coomassie staining as described in the Methods section (Fig. 1a). In order to verify that the soluble E^rns^-R171 thus prepared is indeed monomeric as described for the intracellular protein obtained from BHK-21 cell lysates^36^, we analysed E^rns^ of the strain Ncp7 by SDS-PAGE in the presence or absence of the disulfide reducing agent β-mercaptoethanol (2-ME). Under non-reducing conditions, E^rns^-C171 appeared predominantly as a dimer, whereas the R171 mutant appeared almost completely as a monomer (Fig. 1b). By contrast, both forms of E^rns^, *i.e*., C171 and R171, migrated as a single band at 40–50 kDa in the presence of 2-ME, which indicates the presence of covalently-linked disulfides in E^rns^ C171 (Fig. 1b).

### **Monomeric E**^**rns**^**cleaves ss- and dsRNA*in vitro***

**Figure 2:**
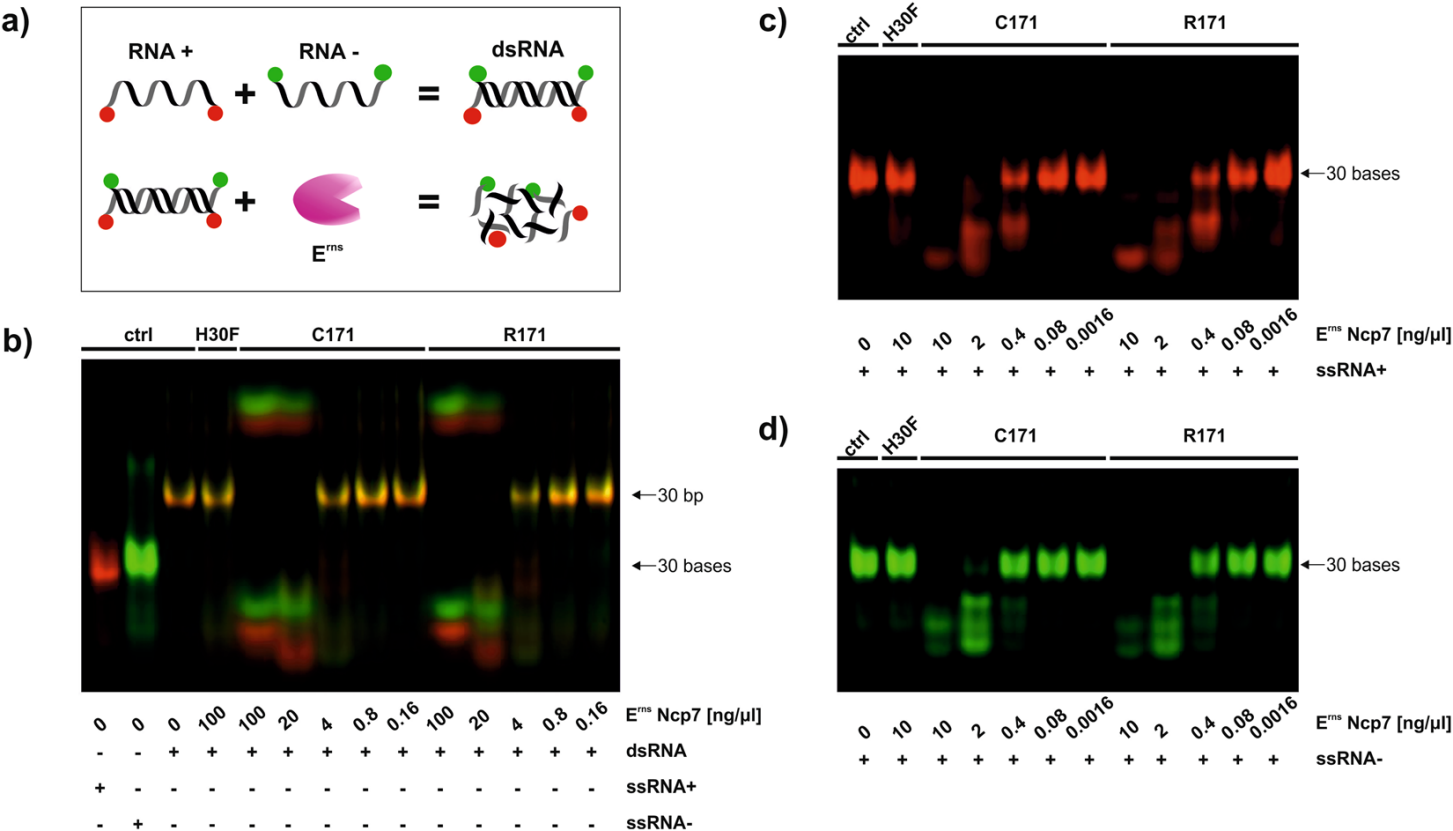
Monomeric E^rns^ cleave ss- and dsRNA *in vitro*. (**a**) Schematic representation of the RNase activity assay with a modified dsRNA fragment. A single-strand RNA of positive (ssRNA+) and of negative polarity (ssRNA−), either labeled in red or green, were boiled and cooled down at room temperature in order to produce the dsRNA fragment. A dilution of Strep-tag purified wild-type (C171), monomeric (R171) and RNase inactive mutant (H30F) of E^rns^ were incubated at the indicated concentrations with 625 nM dsRNA of 30 bp in length (**b**), ssRNA+ of 30 b in length in red (**c**) or ssRNA− of 30 b in length in green (**d**). Samples were separated by 14% PAGE and fluorescence was analysed with a Li-Cor Odyssey system. The identity of the red and green fragments with reduced electrophoretic mobility that appear only upon cleavage remain to be determined. Due to the known, defined length of the directly labeled fragments, no size ladder was applied. Non-cropped gels as representative experiment out of three is shown.

Subsequently, we investigated whether this covalent dimerisation is required to degrade dsRNA. In an *in vitro* RNase assay, E^rns^ Ncp7-C171 and Ncp7-R171 were incubated with a 30 bp dsRNA fragment. Each strand of the dsRNA fragment contained either a red (Dyomics 681) or a green (Dyomics 781) fluorescent dye at their 3′- and 5′-ends (Table 1) to visualize the RNA degradation (see Fig. 2a for a schematic representation). Due to the combination of red and green color, the dsRNA appears as a yellow band in the gel and is thus easily distinguishable from the individual positive- and negative-sense single strands by its color and its different electrophoretic mobility (Fig. 2b, ‘ctrl’ in lanes 1 to 3). Both, wt and monomeric mutant E^rns^ dose-dependently degraded a 30 bp dsRNA fragment with similar efficiency, whereas incubation of the dsRNA with E^rns^ H30F, an RNase inactive mutant, showed no degradation at all (Fig. 2b). Nonetheless, the preference of E^rns^ to degrade single-strand substrates^25,46^ could be confirmed as less E^rns^ was required to completely cleave the corresponding plus- (Fig. 2c) and minus-sense ssRNA (Fig. 2d) from the 30 bp dsRNA fragment analysed. The appearance of small, slow migrating fragments, as e.g. seen in Fig. 2b, could regularly be observed in samples with pronounced RNase activity and might represent the free dye or dye bound to a mononucleotide lacking negatively charged phosphate groups. These slower migrating fragments were also visible upon digestion of ssRNA when overexposing the images. In order to confirm these data, we reassessed the experiment using subgenomic BVD viral dsRNA of 200–300 bp in length as was used in previous experiments^28,47^. In addition to Strep-tag purified E^rns^ of the strain Ncp7 (Supplementary Fig. S1a), we also included non-tagged E^rns^ proteins from the BVDV-I type strain NADL (Supplementary Fig. S1c) in addition to Ncp7 (Supplementary Fig. S1b). As before, dimeric (C171) and monomeric (R171) E^rns^ of both strains equally degraded BVD viral subgenomic dsRNA, whereas the RNase-inactive mutant (H30F) did not cleave the double-stranded substrate (Supplementary Fig. S1). As non-tagged, unpurified E^rns^ still contained unspecific serum RNases as also exemplified in the supernatant of the empty vector control (pCI-SN), RNasin was added that was able to reduce, but not completely eliminate, unspecific RNase activity in the samples as described^28^.

**Table 1:** Sequences of all short nucleotide substrates and their 5′- and 3′-modification by either Dyomics 681 (red) or 781 (green) that were used in the RNase activity assays.

Name	Sequence	3′ and 5′ Modification
ssRNA+	5′-GCC CGU CUG UUG UGU GAC UCG CUC GUC UGC-3′	Dyomics 681
ssRNA−	5′-GCA GAC GAG CGA GUC ACA CAA CAG ACG GGC-3′	Dyomics 781
poly(A)	5′-AAA AAA AAA AAA AAA AAA AA-3′	Dyomics 681
poly(U)	5′-UUU UUU UUU UUU UUU UUU UU-3′	Dyomics 781
poly(C)	5′-CCC CCC CCC CCC CCC-3′	Dyomics 681
poly(G)	5′-GGG GGG GGG GGG GGG-3′	Dyomics 781
metRNA (−)	5′-GCA GAC GAG CGA GUC ACA CAA CAG ACG GGC-3′	Dyomics 781
ssDNA (−)	5′-GCA GAC GAG CGA GTC ACA CAA CAG ACG GGC-3′	Dyomics 781

### **Monomeric E**^**rns**^**cleaves dsRNA*in vitro*under denaturing conditions**

**Figure 3:**
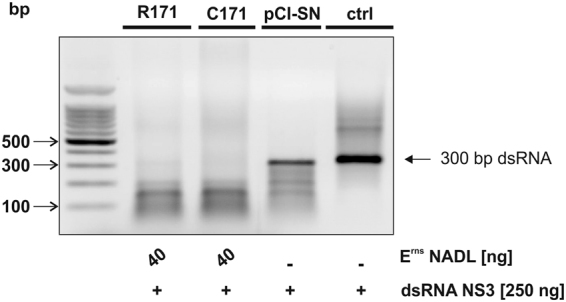
Monomeric E^rns^ cleaves dsRNA in denaturing conditions *in vitro*. Non-tagged C171 and R171 E^rns^ of the BVDV strain NADL were pre-incubated for 5 min in 20 mM sodium citrate buffer pH 6.0 that contained 7.2 M urea and 1 mM EDTA prior to the addition of 250 ng dsRNA of 300 bp in length of the NS3-region of the genome of BVDV strain Suwa. The reaction mixture was incubated for 60 min at 37 °C in a final volume of 8 μl. Medium (ctrl) and the supernatant of the HEK cells transfected with the empty vector (pCI-SN), used at equal volumes as that of the corresponding E^rns^ sample, served as a control for unspecific RNase activity. Finally, the RNA was separated on a 1% agarose gel containing ethidium bromide for 25 min at 100 V and visualized under UV light. One representative experiment out of three is shown.

As E^rns^ enzymes lacking the cysteine at position 171 might still form homodimers by hydrophobic, non-covalent interactions, we investigated whether the RNase retains its capacity to cleave viral RNA under denaturating conditions. Therefore, unpurified E^rns^ NADL-C171 and NADL-R171 (compare Supplementary Fig. S1) were incubated with the RNA substrates in the presence of 7.2 M urea as strong chaotropic agent to remove non-covalent interactions. Nevertheless, both, the monomeric and the covalently-linked dimeric, forms of E^rns^ efficiently degraded a 300 bp fragment of the NS3 (Fig. 3) and the 5′-UTR region (not shown) of the BVD viral genome. As RNasin was not active under these buffer conditions, weak non-specific RNase activity could still be observed with the supernatant of the empty vector control (pCI-SN).

### **Monomeric E**^**rns**^**inhibits dsRNA-induced IFN synthesis**

**Figure 4:**
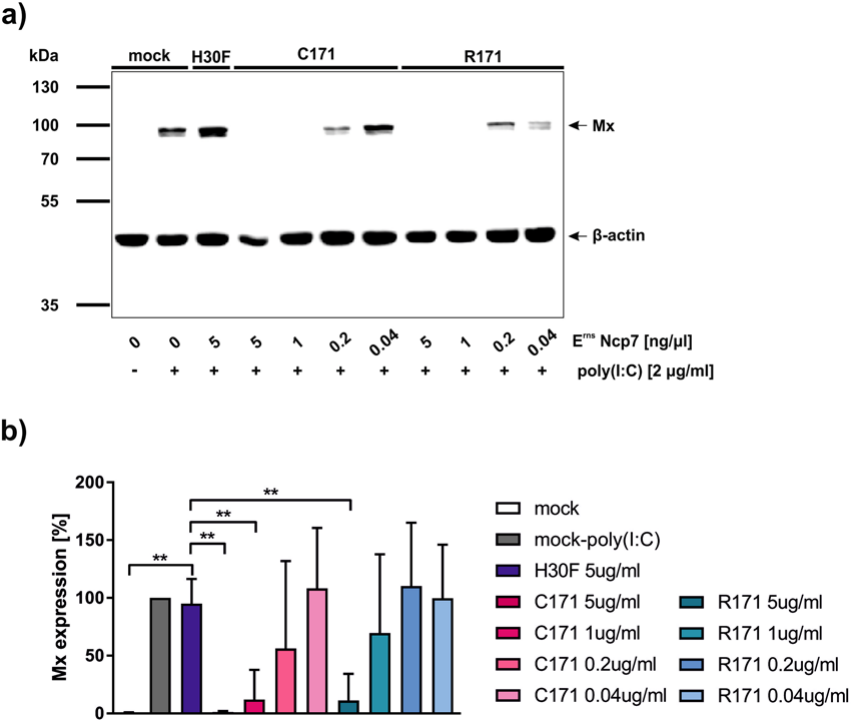
Monomeric E^rns^ inhibits dsRNA-induced IFN synthesis. (**a**) Strep-tag purified wild-type (C171), monomeric (R171) and RNase inactive mutant (H30F) E^rns^ of the BVDV strain Ncp7 were pre-incubated on BT cells for 30 min at the concentrations indicated for 30 min prior to addition of poly(I:C). After 24 h, cells were harvested, cytosolic protein extracts collected and analysed for Mx expression by Western blot (exposure time 10 s). Simultaneous staining of β-actin on the same membrane was used as a control for the protein loading of the individual lanes. PageRuler Plus pre-stained protein ladder was used for size determination. (**b**) Five independent replicates performed as described for panel a were quantified for the signal intensities of Mx expression relative to the expression levels of the corresponding value for β-actin with poly(I:C) induced Mx expression in the absence of E^rns^ set to 100% (mean ± SD, n = 5). All significant differences in the pairwise comparison with the value obtained with inactive E^rns^ (H30F) are indicated with **(p < 0.01).

Others and we previously showed that wt E^rns^ is able to inhibit IFN synthesis induced by extracellularly added ss- and dsRNA of viral origin and by synthetic dsRNA such as poly(I:C)^24,26–28^. In accordance with the *in vitro* results presented above, Strep-tagged wt dimeric (C171) and mutant monomeric (R171) E^rns^ of the BVDV strain Ncp7 significantly inhibited poly(I:C)-induced Mx expression (Fig. 4), a widely used marker for the presence of IFN^2^. Similarly, non-tagged E^rns^ C171 and R171 from both the BVDV strains, Ncp7 and NADL, also displayed equal efficiency in inhibiting poly(I:C)-stimulated Mx expression (Supplementary Fig. S2), further excluding a strain-specific effect.

### **Extended E**^**rns**^**substrate specificity**

**Figure 5:**
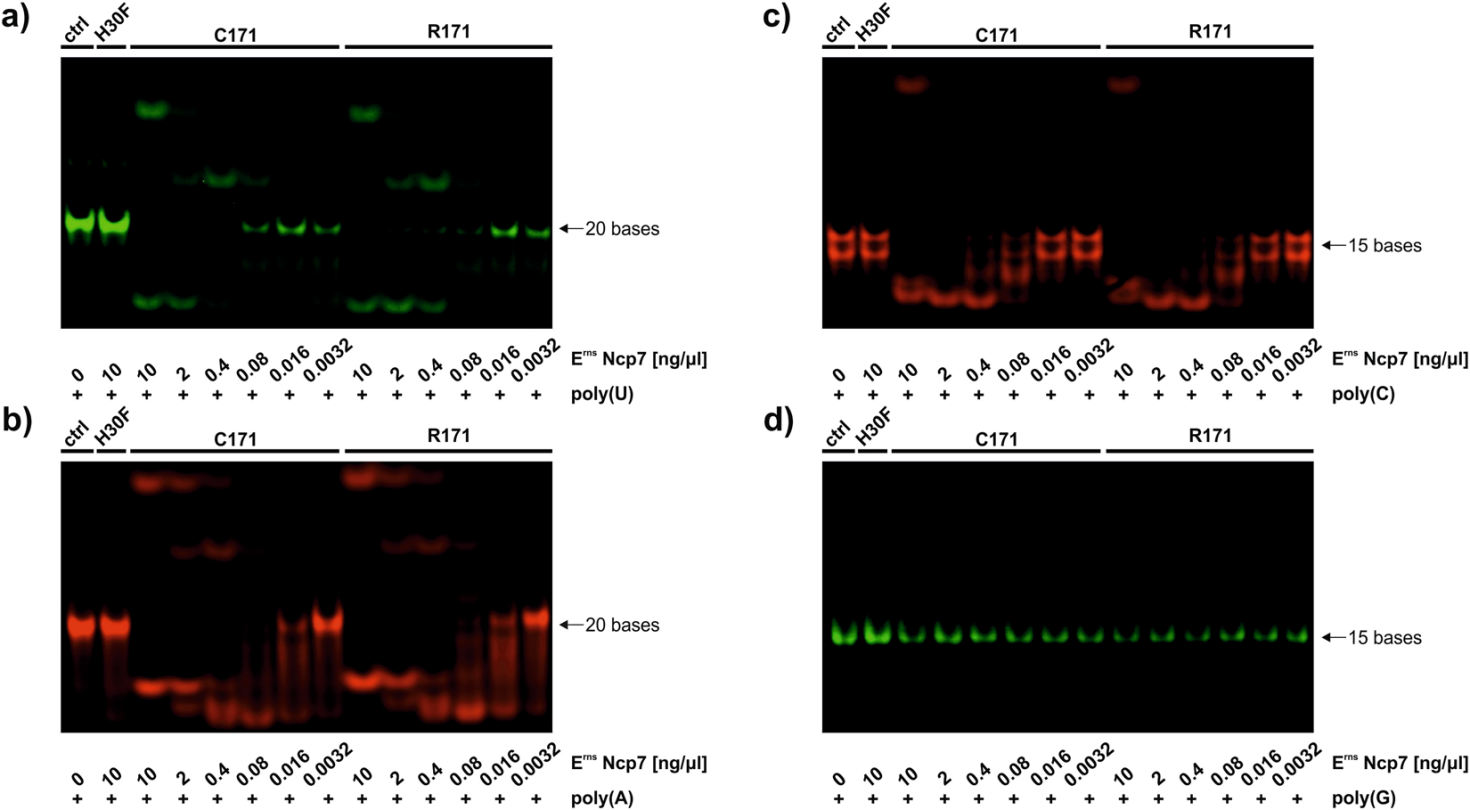
Extended E^rns^ substrate specificity. Various dilutions of Strep-tag purified wild-type (C171), monomeric (R171) and RNase-inactive mutant (H30F) E^rns^ were incubated with 625 nM of poly(U), poly(A), poly(C) and poly(G), either labeled in green or red (panels a–d) as described in Table 1. Samples were separated by 14% PAGE and fluorescence was analysed with an Azure c600 imaging system. Due to the known, defined length of the directly labeled fragments, no size ladder was applied. Non-cropped gels as representative experiment out of three are shown.

E^rns^ belongs to the T2 family of endoribonucleases that preferably degrade ssRNA^25^. It is also known that E^rns^ preferably cleaves RNA prior to uridine residues^25,46,48^. However, poly(I:C)-induced IFN synthesis is clearly inhibited by RNase-active E^rns^^24,26,27^, indicating that the substrate specificity is not absolute. Accordingly, the degradation of the positive (+) and negative-sense (−) ssRNA showed no clearly distinct degradation pattern (Fig. 2), although the ssRNA of negative polarity contains only a single uridine base in contrast to 10 uridines present in ssRNA+ (Table 1). Therefore, we further investigated the substrate specificity of E^rns^. Short RNA polymers of the nucleobases adenine, uridine, cysteine, and guanine (poly(A), poly(U), poly(C), and poly(G), respectively) containing either a red (Dyomics 681) or a green (Dyomics 781) fluorescent dye at their 3′- and 5′-ends were synthesized (Table 1). While poly(A), poly(U) and poly(C) were cleaved by E^rns^, with poly(C) being cleaved less efficiently than poly(U) and poly(A), homopolymeric poly(G) was protected from degradation at up to 10 ng/µl (Fig. 5). Nevertheless, at approx. one order of magnitude higher concentrations of E^rns^, cleavage of poly(G) could be observed (not shown). But on all accounts, the dimeric and the monomeric form of E^rns^ degraded the homopolymeric substrates with similar efficiency (Fig. 5).

### **E**^**rns**^**acts as a nicking endoribonuclease**

**Figure 6:**
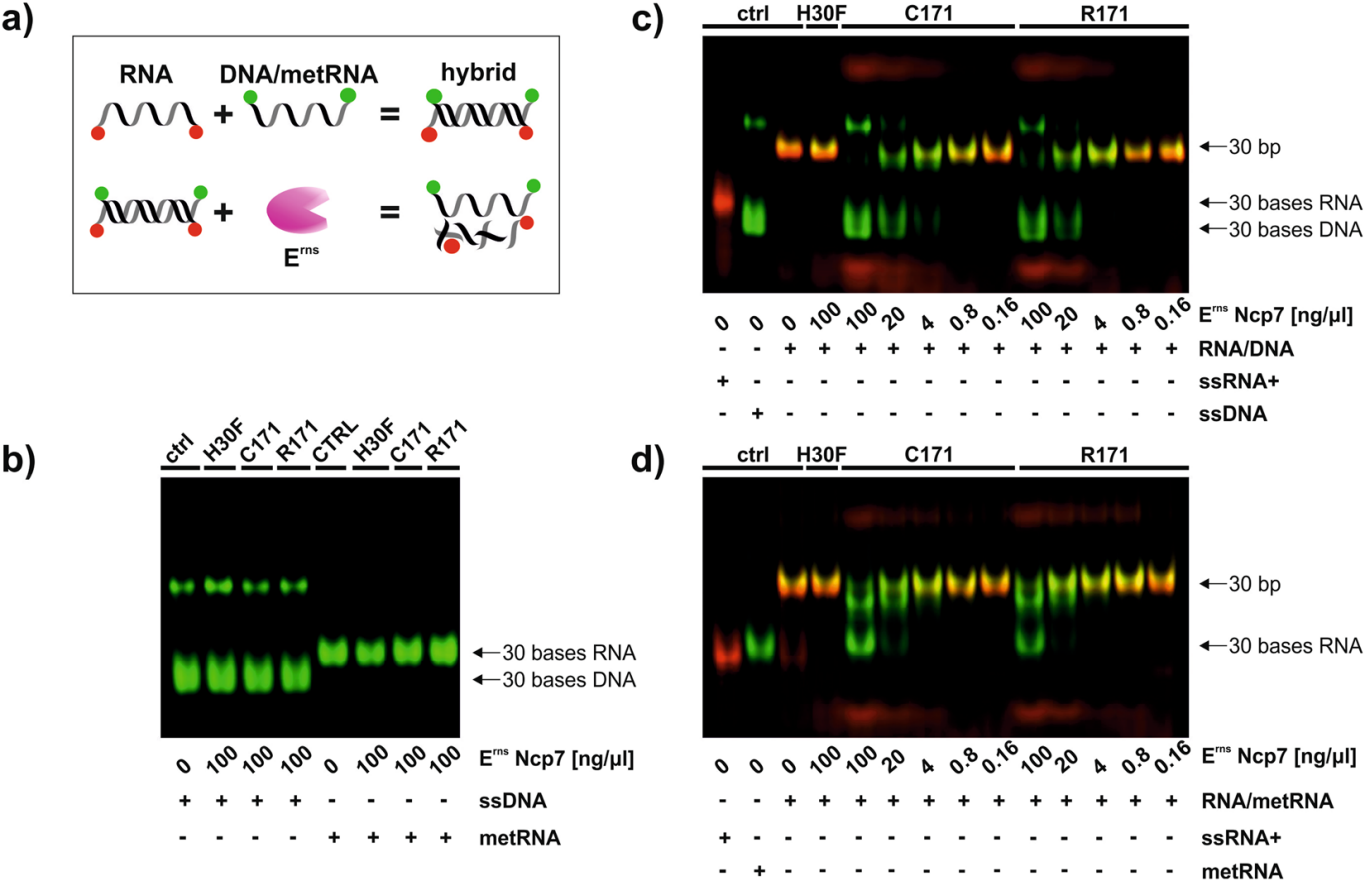
E^rns^ is a nicking endoribonuclease. (**a**) Schematic representation of the RNase activity assay with the modified hybrids. A single-stranded RNA of positive polarity (ssRNA+) labeled in red together with either a single-stranded methylated RNA (metRNA) or a ssDNA of negative polarity, both labeled in green, were boiled and cooled down at room temperature for hybridization (RNA/metRNA or RNA/DNA). Strep-tag purified wild-type (C171), monomeric (R171) and RNase-inactive mutant (H30F) of E^rns^ were incubated at the concentrations indicated with 625 nM single-stranded metRNA or DNA (**b**), double-stranded RNA/DNA− (**c**) or double-stranded RNA/metRNA-hybrids (**d**). Samples were separated by 14% SDS-PAGE and fluorescence was analysed with a Li-Cor Odyssey system. Due to the known, defined length of the directly labeled fragments (30 b for ssRNA, 30 bp for dsRNA), no size ladder was applied. Non-cropped gels as representative experiment out of three (**b**) or four (**c**,**d**) are shown.

According to a crystal structure analysis of the catalytic domain of E^rns^, a dsRNA substrate does not fit into one active site^49^. Nevertheless, we could show that the monomeric mutant of E^rns^ containing just one active site was still able to degrade dsRNA (Fig. 2B). Together with the fact that E^rns^ is preferentially a single strand endoribonuclease, we postulated that E^rns^ might cleave just one strand out of a dsRNA. Therefore, we incubated E^rns^ together with a double-stranded substrate containing an RNA strand hybridized to a complementary strand that is resistant to RNase degradation, *i.e*., 2′-O-methylated RNA (metRNA) or DNA (see Fig. 6a for a schematic representation). Each strand was modified at its 3′- and 5′-ends with a red or green fluorescent dye to be able to differentiate the susceptible (red) from the protected strand (green). The latter was confirmed by the fact that single-stranded DNA and metRNA were resistant to degradation even at the highest concentration of E^rns^ applied (Fig. 6b). Both, dimeric E^rns^ C171 and monomeric E^rns^ R171, degraded the susceptible RNA strand in the RNA/DNA- as well as in the RNA/metRNA-hybrid with the DNA and metRNA strand being resistant to degradation (Fig. 6c,d). Thus, the double-stranded hybrid appears in yellow in the analysis by gel electrophoresis (Fig. 6c,d), which disappears by cleavage of the susceptible RNA (in red) by RNA-active E^rns^. Thereby, the resistant strand (green) is gradually released from the double strand and finally displays the same electrophoretic mobility as displayed by its single stranded control (Fig. 6c,d), including the probably higher order structured form of ssDNA displaying reduced electrophoretic mobility. In order to exclude a role of the buffer composition in the formation of dsRNA, we repeated the experiment in the buffer required for RNase III to exclusively degrade dsRNA (Supplementary Fig. 3). Thus, RNase III degraded dsRNA but none of the other substrates (ssRNA and methylated RNA/RNA hybrid), which confirms the double-stranded form of the corresponding substrates. Nevertheless, E^rns^ was still able to cleave dsRNA and ssRNA either present alone or in the hybrid form. As expected, the RNase-inactive mutant of E^rns^, H30F, did not cleave any substrate, even though some non-specific degradation of the highly susceptible ssRNA by contaminating RNase was occasionally observed (see e.g., right panel in Supplementary Fig. 3).

## Discussion

Over the past years, we and others have demonstrated that BVD viruses efficiently evade their host’s innate immune response. Specifically, the N-terminal autoprotease N^pro^ and the structural protein E^rns^ are viral antagonists of the IFN type-I system that is the most effective innate antiviral defense mechanism of vertebrate hosts. In addition to its location on the surface of virus particles, the viral glycoprotein E^rns^ is secreted from infected cells and inhibits IFN expression induced by ss- and dsRNA by virtue of its RNase activity. Therefore, we proposed that E^rns^ acts as an enzymatically active decoy receptor that degrades any extracellular pestiviral RNA that might act as a PAMP to prevent it from being recognized by PRRs activating the innate immune response (for reviews, see refs^2,5,17,18^).

Besides pestiviruses, only few other viruses in vertebrates were reported to express ribonucleases, e.g. retroviruses^50^, negative-strand RNA viruses^51^, herpesviruses^52^ or the nidoviruses^53^. Mostly, these RNases are required for viral replication but the precise substrates of these RNases are not known. In addition, these RNases are located intracellularly, and only a few such as herpes virus vhs protein^54^ or arteriviral nsp1β^53^ or nsp15 of coronaviruses^55,56^ were reported to be involved in innate immune evasion. The pestiviral RNase E^rns^ belongs to the T2 family of ribonucleases^25^, which mostly consist of a single polypeptide chain and preferably degrade ssRNA^57–59^. Likewise, members of the RNase A superfamily are small polypeptides that are selective for ssRNA except for the aforementioned bovine seminal RNase^42,60^. The latter represents the only member of the RNase A superfamily that naturally exists as a homodimer covalently linked by a disulfide bond and that is active against ss- and dsRNA^42^. However, it was called into question whether the dimeric form is required for dsRNA cleavage, and it was rather proposed that the presence of specific basic charges in an RNase determines the enzymes ability to unwind the double-stranded polyribonucleotide followed by its hydrolysis^60,61^. Thus, monomeric forms of bovine seminal RNase remain active RNases and the cytotoxic activity of the dimer in contrast to the monomer is rather related to its resistance to inhibition by the cytosolic ribonuclease inhibitor protein^41^ than to a different dsRNase activity. By contrast, artificial oligomerisation of members of the RNase A superfamily lead to protein complexes that acquired the property to cleave dsRNA^62^. Similarly, dsRNases such as Drosha or Dicer require homo- or heterodimerisation of their RNase III domains to function (ref.^63^, and references therein). Hence, it was tempting to speculate that E^rns^ requires its dimeric form to gain the ability to cleave single- and double-stranded substrates.

Therefore, we expressed soluble wt and mutant E^rns^ with the latter being unable to dimerise according to the results reported by the group of G. Meyers^36^. In addition to the BVDV strain NADL, we also used the strain Ncp7 to express the viral RNase, as the NADL strain is an ancient strain with a long and poorly documented passage history, and as it is one of the very few strains reported to lack C171^15,36^. Thus, using purified E^rns^ expressed in transiently-transfected HEK293 cells, we similarly observed that wt, but not the mutant E^rns^ C171R, exists as covalently-linked homodimer (Fig. 2). Previously, it was demonstrated that E^rns^ unable to form disulfide-linked dimers, either by mutation of the corresponding cysteine residue or in the presence of high concentrations of thiol-reducing agents, still cleaves ssRNA substrates^25,36,37,48^. In addition, this ssRNase activity is resistant to high salt or urea concentrations^46,48^. Here, we were able to provide strong evidence that homodimerisation of E^rns^ is not required to cleave dsRNA, neither short stretches of synthetic dsRNA (Fig. 2) or *in vitro* transcribed RNA of approximately 300 bp length constructed from viral RNA (Fig. S1). Cleavage of viral RNA also occurred under strong denaturating conditions (Fig. 3) as was shown for the wt enzyme^46^, which confirms that dimerisation by non-covalent, hydrophobic interactions is not required for RNase activity. Finally, the characteristic of E^rns^ to inhibit dsRNA-induced IFN expression was not altered independently of whether the RNase was a monomer or a dimer. Thus, E^rns^ C171 or R171 of the BVDV strains NADL or Ncp7 dose-dependently inhibited poly(I:C)-stimulated Mx expression with similar efficiency (Figs. 4 and S2).

In view of the results presented so far, the mechanism of E^rns^ to cleave dsRNA remains unclear as the recently reported crystallographic structure of the catalytic domain of E^rns^ did not provide evidence for dsRNA to fit into the active site^49^. In analogy, there is a large variety of mechanisms by which restriction endonucleases cleave dsDNA. Thus, classical type-II restriction endonucleases such as EcoRI require homodimerisation to cleave both strands of DNA simultaneously^45^, a way we excluded for E^rns^ in this study. FokI, an unusual type-II restriction endonuclease, is a monomer in solution, but it recruits a second monomer only upon binding to its cognate site on the DNA substrate^64^. However, as E^rns^ is fully functional under strong denaturing conditions, the formation of transient dimers is rather unlikely. Monomeric restriction endonuclease such as BcnI^65^ or dimeric BfiI that possesses only a single active site^66^, cut dsDNA by sequentially nicking the strands individually. Analogously, a monomeric mutant of EcoRI cleaves both strands of DNA independently^67^, a mechanism reminiscent of nicking endonucleases^68^. Thus, it may well be envisaged that E^rns^ independently cleaves both strands of dsRNA – with or without unwinding the dsRNA as discussed above – and the double strand is entirely cleaved into smaller fragments when both nicks in the opposite strands are sufficiently close to each other. This is supported by the fact that it is similarly observed in buffer conditions required for RNase III digestion of dsRNA, confirming the double-stranded nature of the substrates used (Supplementary Fig. 3). This model would be in accordance with the fact that ssRNA is degraded by E^rns^ more efficiently than dsRNA^28^. A similar mechanism that is in agreement with E^rns^ being a nicking endoribonuclease was recently proposed by Krey *et al*. for the cleavage of the ssRNA substrate poly(U)^49^. Thus, cleavage of poly(U) was a multistep process showing a random, non-processive pattern with intermediate dissociation from the enzyme.

In order to support our model of E^rns^ being a nicking endoribonuclease, we designed double-stranded substrates where one of the two strands was resistant to degradation by RNases, *i.e*., by using methylated RNA or DNA. In accordance with our model, monomeric and dimeric E^rns^ were able to degrade a susceptible RNA strand within a double-stranded substrate with the complementary strand being protected from degradation with similar efficiency (Fig. 6). Nevertheless, the degradation of ssRNA substrates was clearly more efficient than that of dsRNA, as higher concentrations of the viral RNase were required to degrade double-stranded polynucleotides to a similar degree. Thus, it might be envisaged that cleavage of dsRNA prevents activation of TLR-3^47^ whereas the high efficiency of ssRNA degradation to very small fragments impedes activation of TLR-7/8 by guanosine and uridine combined with short ssRNA, respectively^69^.

Despite the fact that we demonstrated that the monomeric form of E^rns^ is equally effective in cutting dsRNA as the dimer, the cysteine at position 171 is conserved in evolution in the majority of pestivirus strains analysed to date, and infectious viruses with mutant E^rns^ are clearly attenuated *in vivo*^36^. However, E^rns^-mutant viruses were also slightly reduced in their replication efficiency^36,37^ that might be caused by an altered affinity for its substrate of the RNase monomer compared to the dimer^49,70^. Nonetheless, it has to be kept in mind that even small differences in fitness might have considerable deleterious consequences for a given virus after numerous passages *in vivo* or *in vitro* (for review, see e.g. ref.^71^). In addition, the fact that mutant viruses coding for monomeric E^rns^ are attenuated might be independent E^rns’^ ability to evade the host’s IFN response. For instance, the thermodynamic stability of the monomer might differ from that of the dimer influencing the efficiency of protein secretion^72^, although no such effect was seen for E^rns^ expressed in transiently transfected BHK-21 cells^36^. Otherwise, oligomerisation of E^rns^ might influence the assembly or budding of the virus or the stability of the virus particle^73^. Accordingly, amphipathic helixes, as present in the C-term of E^rns^ ^31^, are known to generate positive curvature in lipid membranes^74,75^, which might be required for efficient virus particle formation.

It remains somewhat of a surprise that an enzyme can degrade structurally diverse substrates such as ss- and dsRNA. However, the specific substrate specificity of E^rns^ is not yet known, and the precise intra- or extracellular site of its action and the identity of the PRRs that it is able to block is of great interest. Given the small genome size of many RNA viruses, many proteins encoded by these viruses fulfill several functions, and therefore, it is highly probable that E^rns^ performs tasks other than its RNase activity, *e.g*., as described for the single human RNase T2 protein^76^. Nevertheless, we provide strong evidence that homodimerisation of E^rns^ is not required for degradation of dsRNA and for inhibition of dsRNA-induced IFN synthesis in cultured cells. However, it may well be that oligomeric E^rns^ has functions different from the monomeric form that is capable of cleaving dsRNA *in vitro*. Therefore, the role of the evolutionary conserved dimerisation of E^rns^, either as a viral envelope glycoprotein or as secreted RNase, remains to be identified, and it has to be kept in mind that different cells or organs are differentially regulated to comply with their specific need^77,78^.

## Methods

### Cells and Reagents

Cell culture media were purchased from Biochrom (Bioswisstec AG, Schaffhausen, Switzerland), and fetal calf serum (FCS) was obtained from PAA Laboratories (Lucerna-Chem AG, Lucerne, Switzerland). FCS was free of BVDV and antibodies to BVDV as tested by virus isolation and serum neutralisation assays, respectively.

Primary bovine turbinate (BT) cells were prepared from fetuses which were collected from a local abattoir. Madin-Darby Bovine Kidney (MDBK) cells were obtained from ATCC. Cells were maintained in Eagles minimal essential medium (MEM) containing 7% FCS during the experiments, penicillin (100 IU/ml) and streptomycin (100 μg/ml) at 37 °C in a humidified 5% CO_2_ atmosphere. All cells were found to be free of BVDV by immunoperoxidase staining. The HEK 293 T/17 cells were kindly provided by P. Plattet (Division for Experimental Clinical Research, Vetsuisse Faculty, University of Bern), and were propagated in Dulbecco’s MEM (DMEM) with 10% FCS and penicillin/streptomycin as indicated above.

Restriction enzymes were obtained from Fermentas (Thermo Scientific, Wohlen, Switzerland). GoTaq DNA Polymerase, BenchTop 100 bp DNA ladder and RNasin were from Promega, whereas the RNA loading dye was obtained from New England Biolabs (Ipswich, MA).

### Expression of non-tagged E^rns^

Non-tagged wt E^rns^ from the BVDV strains Ncp7^79^ and NADL (the prototype cp BVDV strain^80^) was originally expressed in MDBK Tet-On cells using a tetracycline-inducible expression plasmid as described^27^. The E^rns^ obtained from the BVDV strain Ncp7 contains a cysteine residue at position 171 (C171), whereas the E^rns^ of strain NADL was cloned out of a cDNA (kindly provided by C. Rice, Rockefeller University, New York, USA) encoding for an arginine at this position (R171). The corresponding mutants Ncp7-R171 and NADL-C171 were generated by applying the QuikChange II XL Site-Directed Mutagenesis Kit (Stratagene; Agilent Technologies AG, Basel, Switzerland).

To be more efficient at producing various mutants of E^rns^, we switched to protein synthesis in HEK cells transiently transfected with the corresponding expression plasmids. For this purpose, standard PCR techniques were employed (primers are available upon request) to amplify and clone E^rns^ from the MDBK Tet-On cells, and ligated into the pCI mammalian expression vector (Promega). The inserted sequences in every plasmid were verified by nucleotide sequencing. To express the various forms of E^rns^, HEK cells were transfected with the corresponding pCI-E^rns^ or the empty vectors using Fugene HD (Roche Diagnostics, Rotkreuz, Switzerland) at a transfection reagent to cDNA ratio of 3:1, according to manufacturer instructions, followed by 3 days-incubation. Supernatant was harvested, filtered through 1-MDa size-exclusion Vivaspin columns (Milian AG, Wohlen, Switzerland), and concentrated by ultrafiltration using 10-kDa size-exclusion Vivaspin columns (Milian). The concentration of E^rns^ was determined by ELISA as described above, and the activity of E^rns^ expressed in bovine MDBK cells was equivalent to that expressed in human 293T-cells as analysed by their ability to dose-dependently inhibit Mx expression in BT cells induced by poly(I:C) (Sigma, Buchs, Switzerland) as described^27,28^.

### Expression of Strep-tagged E^rns^

The sequence for wild-type (wt) E^rns^ of the BVDV strain Ncp7 containing a Twin-Strep-tag (SAWSHPQFEKGGGSGGGSGGSWSHPQFEK) at the C-terminus was codon-optimized, synthesized and subcloned into the pCI mammalian expression vector (Promega; kindly provided by P. Plattet) by Eurofins Genomics GmbH (Ebersberg, Germany). The monomeric mutant that contains an arginine instead of a cysteine at position 171 (R171) and the RNase-inactive mutant H30F^27^ of the strain Ncp7 were subcloned using the “In-Fusion HD cloning kit” from Clontech (Takara Bio Company). Transfection of the plasmids into HEK293 cells and cultivation for 7 days in Freestyle 293 medium (GIBCO, Life Technology) in the presence of valproic acid was done at the “Protein Expression Core Facility” (PEGF) of the Swiss Federal Institute of Technology (EPFL) in Lausanne, Switzerland. The E^rns^ protein present in the HEK cell supernatant was Strep-tag purified using a gravity flow Strep-Tactin Superflow column (IBA GmbH, Göttingen, Germany). Columns were washed five times with wash buffer containing 100 mM Tris/Cl pH 8.0, 150 mM NaCl and 1 mM EDTA, and the Strep-tagged E^rns^ proteins were eluted in six elution steps with wash buffer containing 2.5 mM desthiobiotin (IBA GmbH). The pH was reduced to 7 using 1 M HCl, and the concentration of purified E^rns^ was determined by ELISA as described^27^. The purity of the eluted protein was confirmed by SDS-PAGE under non-reducing condition and Coomassie staining (Coomassie Brilliant Blue G 250; Bio-Rad Laboratories, Cressier, Switzerland).

### RNase assays

Strep-tag purified E^rns^ proteins and RNase III (Thermo Fisher Scientific) were diluted in elution buffer at pH 7 (IBA GmbH) and the oligonucleotide substrates in 100 mM Tris/acetate buffer at pH 5.5 or for experiments shown in Supplementary Fig. S3, in RNase III buffer containing 5 mM NaCl, 1 mM Tris/Cl and 1 mM MgCl_2_ at pH 5.5. DsRNA or hybrids were obtained by incubating together the single strands at 95 °C for 5 min followed by slowly cooling down to ambient temperature. Long viral dsRNA of the 3′- or 5′-region of BVDV was prepared as described^47^, whereas all short oligonucleotides were synthetized and labeled by Microsynth (Balgach, Switzerland). Sequences of all short substrates and their 5′- and 3′-modification by either Dyomics 681 (red) or 781 (green) that were used in the RNase activity assays are shown in Table 1. The dyes are coupled to 3′-and 5′-ends of the short oligonucleotides via an amino-C6 linker to the phosphate. According to Microsynth, homopolymeric oligonucleotides could not be synthesized longer in size as shown in Table 1 for technical reasons. The degradation by E^rns^ of ss- or dsRNA was analysed by incubating the prediluted E^rns^ and substrates at equal volumes for 30 min (single strands) or 90 min (double strands) at 37 °C in a final volume of 10 μl. After digestion, the reaction was cooled on ice, 2x RNA loading dye (NEB; Bioconcept, Allschwil, Switzerland) was added and the samples were boiled at 95 °C for 5 min. Finally, the RNA was separated on a 14% polyacrylamide gel for 1.5 h at 100 V and analysed with an Odyssey (LI-COR Biosciences GmbH, Bad Homburg, Germany) or Azure c600 (Azure Biosystems, Inc.; Axon Lab AG, Baden, Switzerland) imaging system. Long viral dsRNAs were analysed by 1% agarose gel electrophoresis for 50 min at 100 V, and ethidium bromide staining was visualized by UV light with the U:Genius gel imaging system (Syngene; Labgene Scientific SA, Châtel-St-Denis, Switzerland).

### Western blotting

For the expression analysis of the cellular protein Mx, standard Western blotting was used as described^28,81^ apart from using the WesternBright ECL-HRP Substrate (advansta Inc.; Witec AG, Luzern, Switzerland) as chemiluminescence substrate for the detection by the CCD camera (Fujifilm LAS3000). Where applicable, signal intensities were quantified using AIDA (raytest, Straubenhardt, Germany) and statistically analysed. Thus, the different treatments were compared to the reactions of the RNase-inactive mutant E^rns^-H30F used as a control, which was not significantly different from the poly(I:C) positive control (mock-poly(I:C)) set to 100% in each test, using One-Way Analysis of variance (ANOVA) for planned comparisons implemented in NCSS (NCSS LLC, Kaysville UT). For the analysis of E^rns^ monomers or dimers, SDS-PAGE was used. Thus, the protein samples were mixed with 50 mM Tris buffer pH 6.8, 4% SDS, 12% glycerol and 0.01% Brilliant Blue G (Sigma) with or without 2% β-mercaptoethanol (2-ME) for reducing and non-reducing conditions, respectively. The samples were separated on a 10% gel and transferred to nitrocellulose membranes and standard Western blot was performed as described above using a mouse monoclonal antibody^27^ (50F4–10; kindly provided by T. Rümenapf, Institute of Virology, University of Veterinary Medicine, Vienna, Austria) at a dilution of 1:10 (non-reducing conditions) or an anti-Strep-tag mouse monoclonal antibody conjugated with HRP (IBA GmbH) at a dilution of 1:2500. For estimation of the molecular weight of the proteins, either the BenchMark™ pre-stained protein ladder from Life Technologies (LuBioScience GmbH, Lucerne, Switzerland) or PageRuler Plus pre-stained protein ladder (Thermo Fisher Scientific) were used.

## Electronic supplementary material

Supplementary Information
