# Supplementary material for: Homodimerisation-independent cleavage of dsRNA by a pestiviral nicking endoribonuclease
Source: Sci Rep. 2018 May 29;8:8226. doi: 10.1038/s41598-018-26557-4 (PMC5974291; doi:10.1038/s41598-018-26557-4)
Supplement: Supplementary file 1 — Supplementary Information [file 41598_2018_26557_MOESM1_ESM.pdf]

Supplementary Information

Homodimerisation-independent cleavage of dsRNA by a pestiviral nicking endoribonuclease

Carmela Lussi[1,2,3], Kay-Sara Sauter[1,4] and Matthias Schweizer[1,2]

[1] Institute of Virology and Immunology, Laenggass-Str. 122, CH-3001 Bern, Switzerland
[2] Department of Infectious Diseases and Pathobiology, Vetsuisse Faculty, University of Bern, Switzerland.
[3] Graduate School for Cellular and Biomedical Sciences, University of Bern, Switzerland
[4] Current address: Department of Clinical Research, Faculty of Medicine, University of Bern, CH-3010 Bern, Switzerland

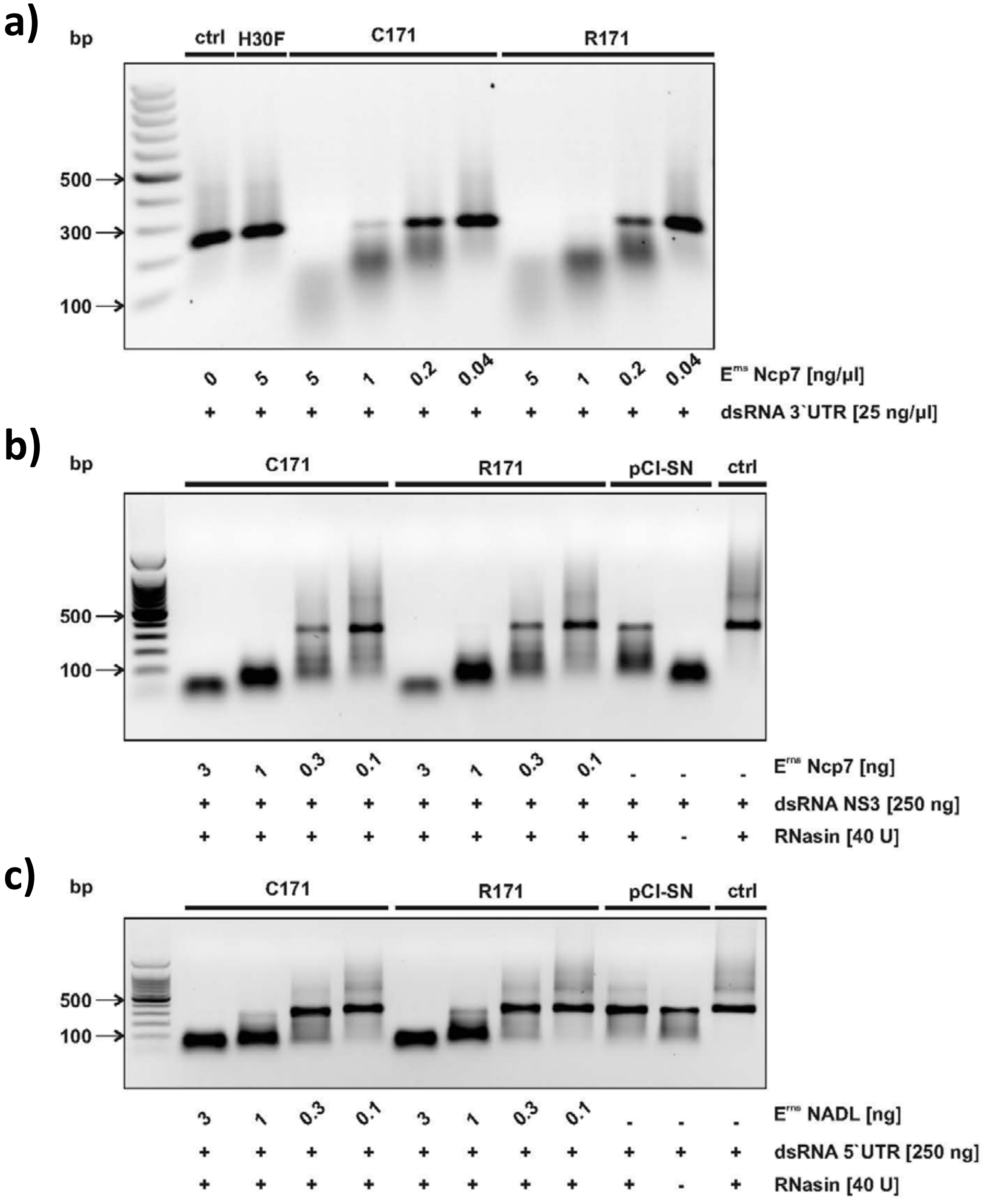

Suppl. Fig. 1. Monomeric E^{rns} cleave ss- and dsRNA *in vitro*

(a) In vitro-transcribed 200 bp dsRNA of the 3'-UTR of the BVDV strain Suwa diluted in 100 mM Tris-acetate buffer at pH 5.5 and Strep-tag purified E^{rns} proteins diluted in elution buffer pH 7 were mixed together at equal volumes to yield a final volume of 10 µl with the concentrations as indicated. After one hour at 37°C, the reaction was analysed by 1% agarose gel electrophoresis and ethidium bromide staining. In the first well, 100 bp DNA ladder (Promega) was loaded as size marker.

(b, c) Non-tagged C171 and R171 E^{rns} of the BVDV strains Ncp7 (b) and NADL (c) were pre-incubated at the indicated concentrations for 5 min with or without 40 U RNasin in 100 mM Tris-acetate buffer pH 6.5 prior to the addition of dsRNA of the NS3- (panel b) or 5'-UTR-region (panel c) of the Suwa BVDV genome. Samples were incubated for 60 min at a final volume of 5 µl at 37°C. Medium (ctrl) and supernatant of the HEK cells transfected with the empty vector (pCI-SN), used at equal volumes as that of the corresponding E^{rns} sample, served as a control for unspecific RNase activity. The RNA was analysed by agarose gel electrophoresis. One representative experiment out of three is shown.

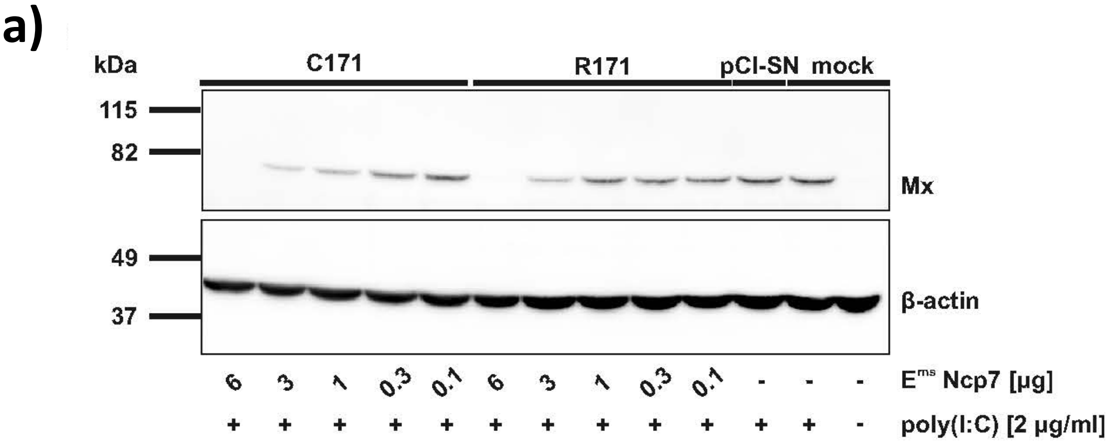

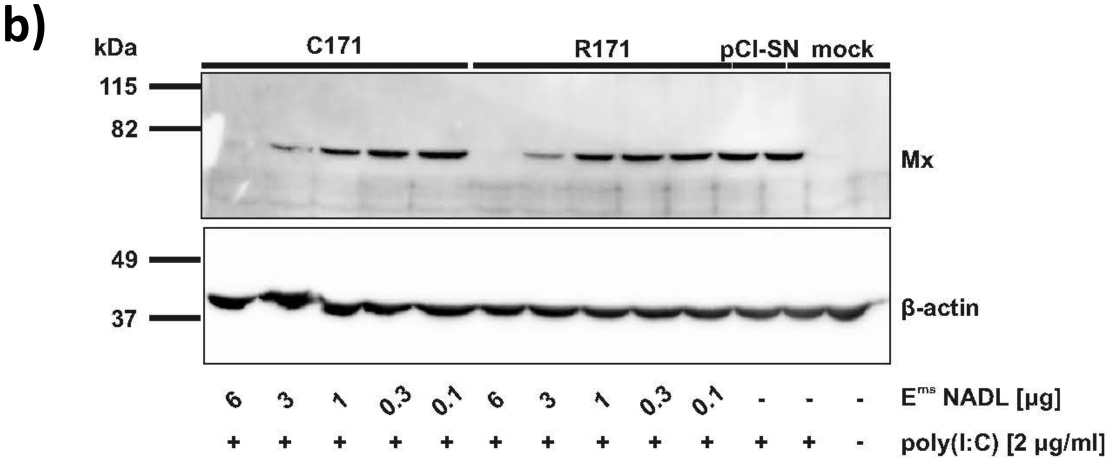

Suppl. Fig 2. Monomeric E^{rns} of the BVDV strains Ncp7 and NADL inhibit dsRNA-induced Mx synthesis.

Non-tagged dimeric (C171) or monomeric (R171) E^{rns} of the BVDV strain Ncp7 (a) and NADL (b) were added at the indicated concentrations with 2 µg/ml poly(I:C) in medium containing 7% FCS to BT cells for 24 h. Cells were harvested, cytosolic protein extracts were collected and analysed for Mx expression (exposure time 1 min) by Western blot. Simultaneous staining of β-actin (exposure time 10 s) on the same membrane was used as a control for the protein loading of the individual lanes. Medium (mock) and supernatant of the HEK cells transfected with the empty vector (pCI-SN), employed at the highest volume as used for one of the E^{rns} samples, served as a control for unspecific RNase activity. BenchMark™ pre-stained protein ladder was used for protein size determination. Representative results out of four (panel a) or three (panel b) experiments are shown.

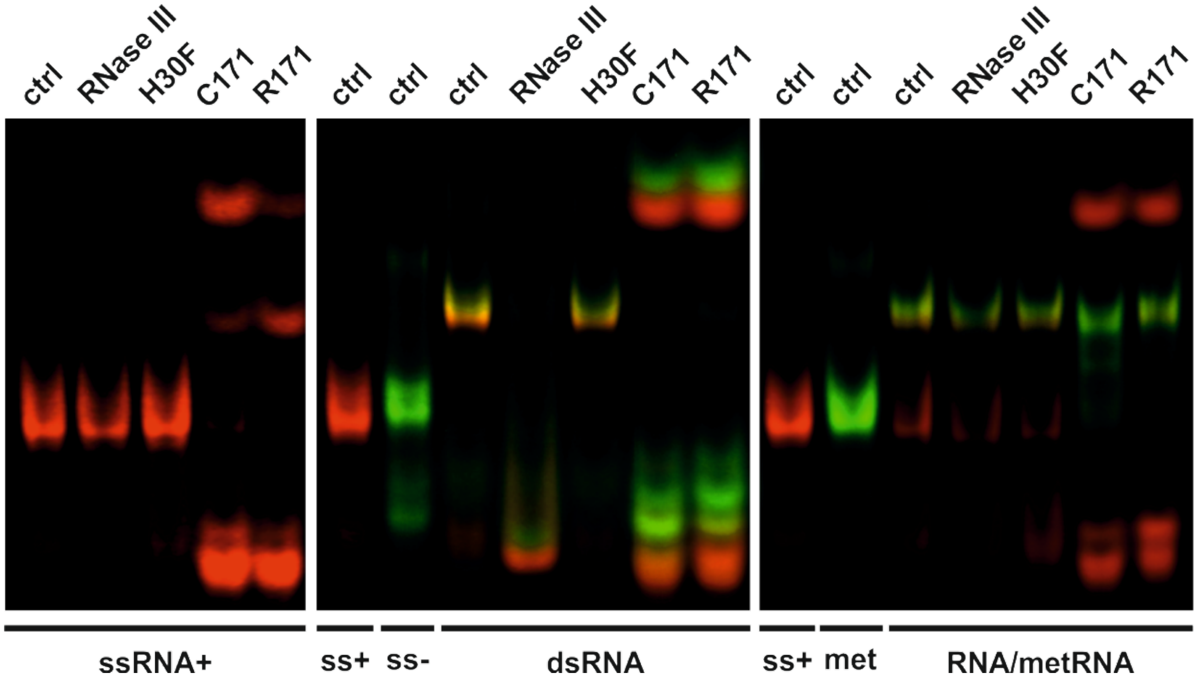

Suppl. Fig 3. E^{rns} and RNase III degrade dsRNA, but only E^{rns} cleaves RNA in a double-stranded hybrid.

Fluorescently labeled positive-sense single-strand RNA (ssRNA+ or ss+; red), negative-sense single-strand RNA (ssRNA- or ss-; green), double-strand RNA (dsRNA) and methylated RNA (green)/RNA+ hybrids of 30 b and 30bp in length, respectively, were incubated for 30 min (ssRNA+) or 1.5 h (dsRNA and RNA/metRNA) together with RNase III (0.5 U per reaction) or wild-type or mutant E^{rns} of the BVDV strain Ncp7. Strep-tag purified wild-type (C171), monomeric (R171) and RNase-inactive mutant (H30F) of E^{rns} were used at the highest concentrations corresponding to Fig. 2c (ssRNA+), Fig. 2b (dsRNA) and Fig. 6d (RNA/metRNA). Samples were separated by 14% SDS-PAGE and fluorescence was analysed as described in the Methods section. Due to the known, defined length of the directly labeled fragments and the lack of an appropriate marker, no size ladder was applied.

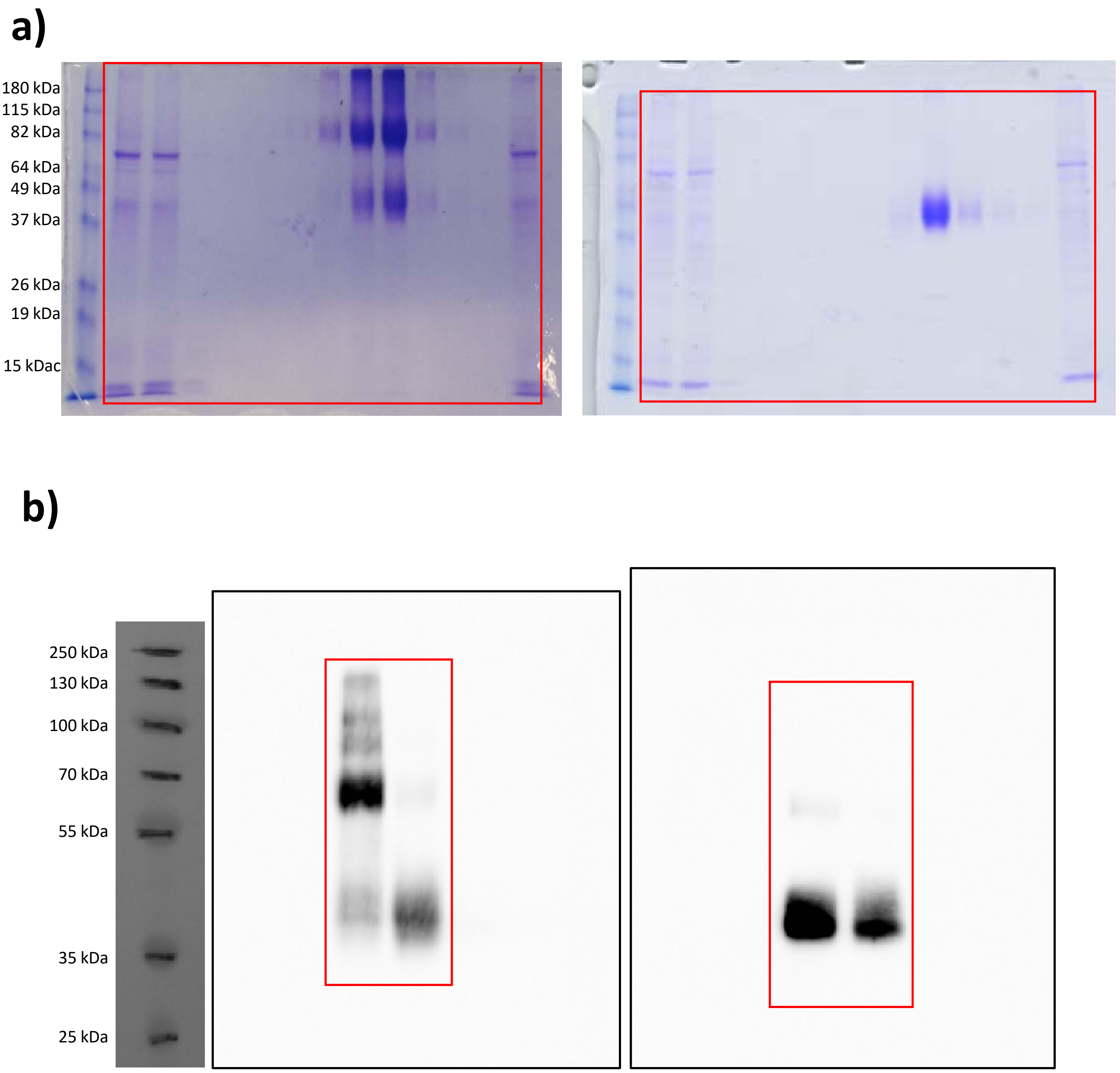

Supplementary Figure 4. Full-length uncropped gels and blots for figure 1.
Cropped areas are marked with red boxes.

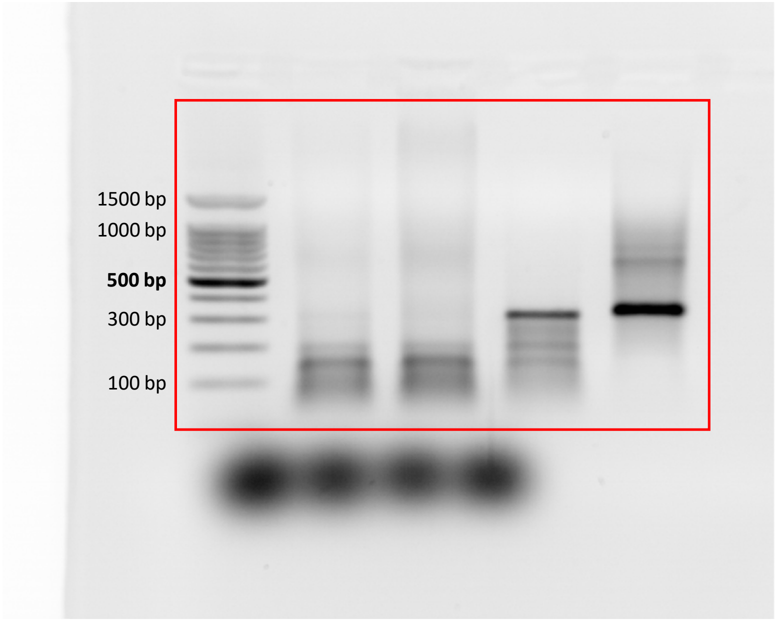

Supplementary Figure 5. Full-length uncropped gel for figure 3.
The cropped area is marked with a red box.

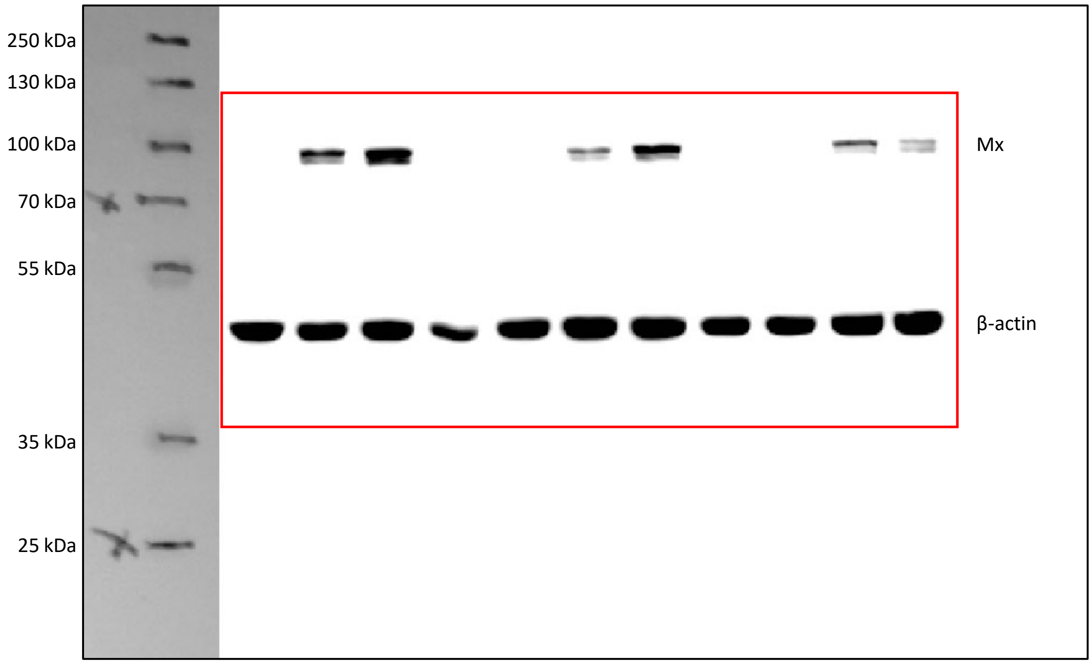

Supplementary Figure 6. Full-length uncropped blot for figure 4.
The cropped area is marked with a red box.

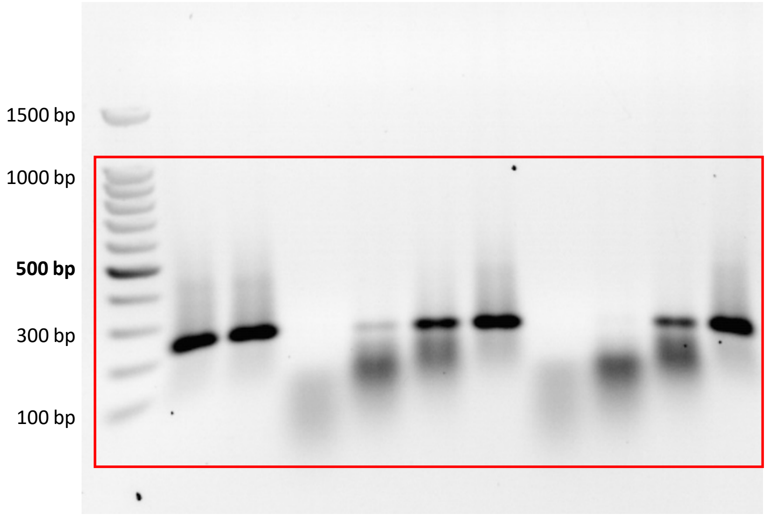

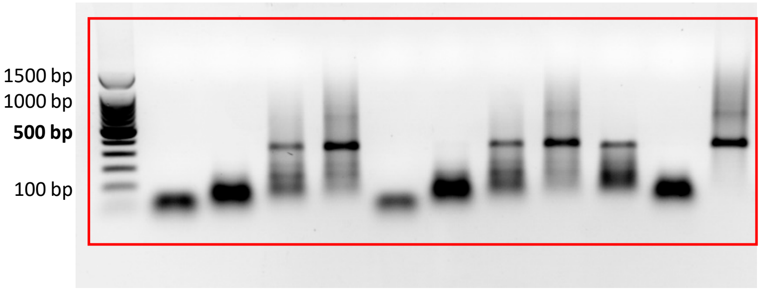

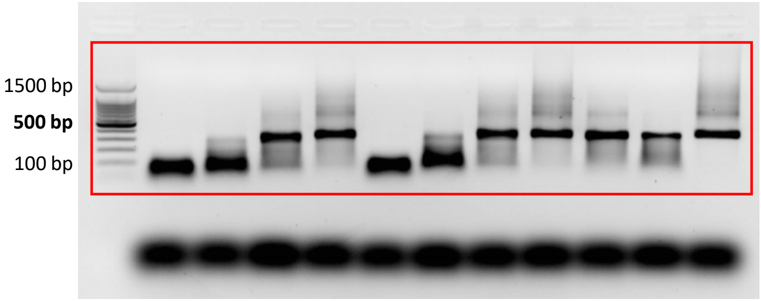

Supplementary Figure 7. Full-length uncropped gels for Supplementary figure 1. Cropped areas are marked with red boxes.

a)

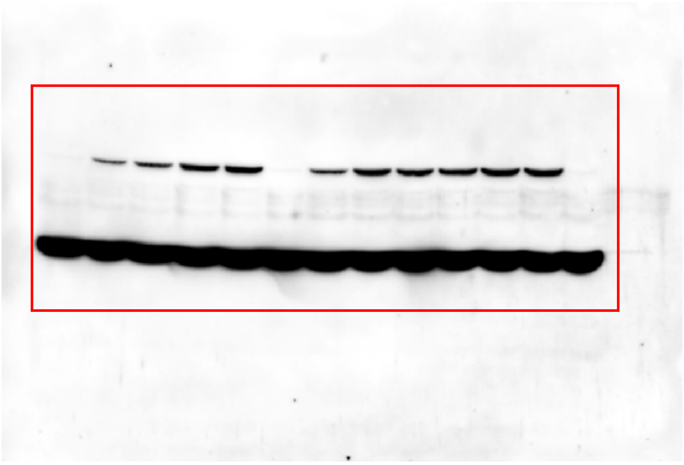

b)

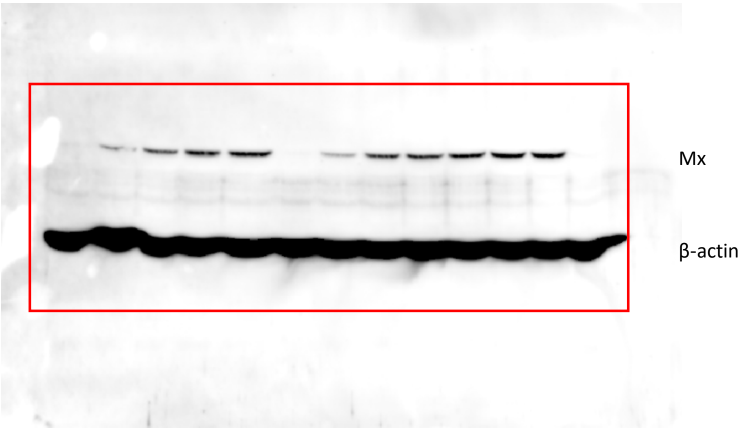

Supplementary Figure 8. Full-length uncropped blots for Supplementary figure 2. In order to display the simultaneous staining for Mx and β-actin, only one single blot with the exposure time of 1 min is shown for each panel. Cropped areas are marked with red boxes.
